# Oocyte Morphology and Reproductive Outcomes - Case Report and Literature Review

**DOI:** 10.5935/1518-0557.20210001

**Published:** 2021

**Authors:** Raquel Meirelles Guimarães, Larissa Maciel Ribeiro, Lizandra Paravidine Sasaki, Hitomi Miura Nakagawa, Iris Oliveira Cabral

**Affiliations:** 1 Genesis - Human Reproduction Assistance Center - Brasílila, DF, Brazil; 2 Taguatinga Regional Hospital - Brasília, DF, Brazil; 3 Maternal-Infant Hospital of Brasília - Department of Human Reproduction - Brasília, DF, Brazil; 4 University Hospital of Brasília - Brasília, DF, Brazil

**Keywords:** oocyte quality, oocyte morphology, oocyte dysmorphism, *in vitro* fertilization (IVF), reproductive outcomes

## Abstract

Oocyte quality could be negatively affected by many factors including smoking, alcohol consumption, obesity, woman’s age, endometriosis and controlled ovarian stimulation (COS), during assisted reproductive technology (ART), in addition to genetic factors, such as hormone receptor polymorphisms, for example. We know that the increase in the reactive oxygen species (ROS) due to systemic disorders causes biochemical and morphological changes to the oocytes, interfering with their quality. The oocyte dysmorphism can be expressed through intra and/or extra cytoplasmic changes. In general, the size and number of oocytes’ morphological abnormalities are directly related to preimplantation development failure. This case report is based on four *in vitro* fertilization (IVF) cycles performed by a patient with oocyte dysmorphism in all oocytes captured. The literature review on this topic aims to relate the characteristics of the oocytes, presented in the case report, with research results about the quality and morphology of the oocytes.

## INTRODUCTION

From the gametes involved in the human reproduction process, the oocyte seems to have a primary role in embryo and fetal outcomes, since it’s there that nuclear, cytoplasmic and functional reorganizations occur, and provides the aggregation of sperm’s genetic material and zygote formation (Keefe *et al.* 2015; Vollenhoven & Hunt, 2018). Oocyte quality can be negatively affected by several factors, including lifestyle, such as smoking and alcohol consumption (Vollenhoven & Hunt, 2018; Prasad *et al*., 2016), obesity (Broughton & Moley, 2017), advanced woman’s age (Vollenhoven & Hunt, 2018; Prasad *et al*., 2016; Keefe *et al*. 2015; Figueira *et al*., 2015), endometriosis (Ceviren *et al*., 2014; Kasapoglu *et al*., 2018; Sanchez *et al*., 2017; Figueira *et al*., 2015) and controlled ovarian stimulation (COS), in assisted reproduction technology (ART) cycles (Figueira *et al*. 2015; Bosch *et al*., 2016), in addition to genetic factors, such as hormone receptor polymorphisms, for example (Alviggi *et al*., 2018; Čuš *et al*., 2019).

We know that a high percentage of oocytes results in incompetent embryos or implantation failure, even though they are morphologically and chromosomally normal. We believe that the energy supply is the main responsible for this fact (Meldrum *et al*., 2016). The exacerbated production of reactive oxygen species (ROS), due to inadequate energy supply, leads to an imbalance between oxidative free radicals and antioxidant substances, biochemically interfering with oocyte development and maturation, causing morphological and functional changes to the egg, and worst outcomes (Vollenhoven & Hunt, 2018; Prasad *et al*., 2016; Keefe *et al*. 2015). Against the limitation of oocyte biochemical analysis in daily clinical practice, a deeper understanding of the morphological aspects of oocytes that could negatively affect reproductive outcomes becomes important (Keefe *et al*., 2015; Setti *et al*., 2011), beyond viability, since oocyte analysis was already part of clinical practice before ART procedures (Kasapoglu *et al*., 2018).

## CASE REPORT

R.S, female, 36 years-old, married, self-employed, with no previous pregnancy. The patient presented with conjugal infertility at the first medical appointment, after a year and a half of attempts to conceive. History of hyperprolactinemia using 0.5 mg Cabergoline/weekly, started two years ago, due to complaints of vaginal dryness. She had a suspected adenomyosis on routine transvaginal ultrasound in the same year. In the initial infertility investigation, her physician ordered hysterosalpingography (HSG), transvaginal ultrasonography (TVUS) for antral follicle count (AFC), hormonal tests to assess ovarian reserve, spermogram, couple’s karyotype, and TVUS for mapping endometriosis.

HSG, spermogram and the couple’s karyotype were within the normal range. Results of the blood tests on 02/08/2017 (3rd day of the cycle): TSH: 1.82 T4l: 1.11 LH: 5.25 FSH: 9.6 E2: 39.1 PRL: 4.8, all of them within the normal range. Results from the TVUS for endometriosis mapping on 12/11/2017: uterus volume of 55.4 cm^3^, endometrium 4.3 mm, myometrium with diffuse hyperechogenic points suggestive of adenomyosis, right ovary (RO) with 7.0 cm^3^ and left ovary (LO) with 3.6 cm^3^ with diffuse hyperechogenic points suggestive of endometriosis. Irregular hypoechogenic lesion without Doppler flow in the left round ligament, juxtaposed to the LO, measuring 9x7x7mm, which may correspond to a deep endometriosis lesion. Results from the TVUS for AFC on 01/09/2018: uterus volume of 48.7cm^3^, endometrium of 4.8mm, RO of 4.0cm^3^, with 04 antral follicles (AF) and residual corpus luteum of 10x6mm, and LO of 5.7cm^3^, with 10 AF and the presence of diffuse hyperechogenic points of 4x3mm and 2x1mm, which may correspond to an indirect sign of endometriosis. In view of the diagnosis of endometriosis and infertility duration, we chose IVF. IVF cycles started according to the following steps.

**Figure 1:**
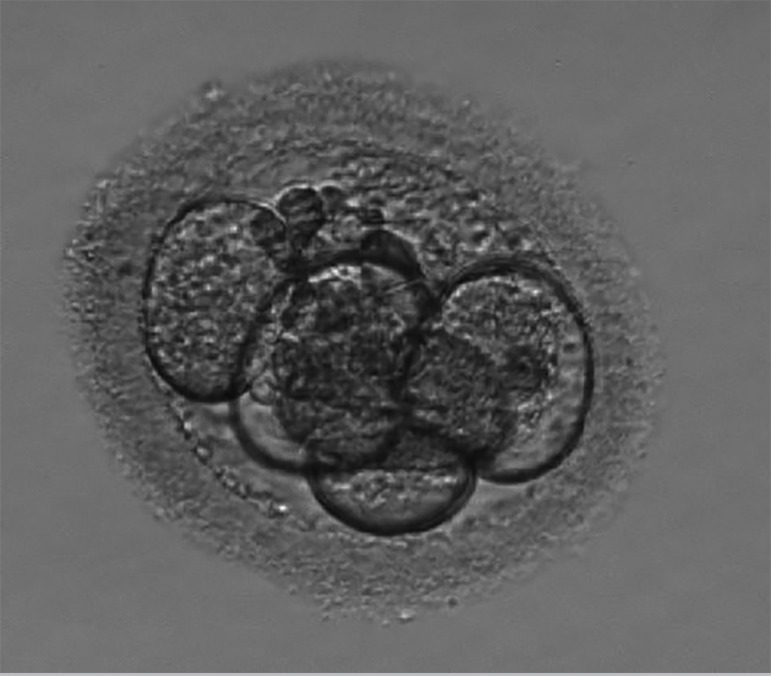
Embryo on the 2^nd^ day of development (1^st^ IVF cycle).

The first IVF cycle occurred in January 2018. The number of antral follicles on the 2^nd^ day of the cycle were 14. We used Leuprolide Acetate 3.75mg intramuscular (IM), single dose on the 21^st^ day of the previous cycle, for pituitary block. Alfa pholithropine/Lutropin Alfa (150UI/75UI) one vial and Alfa pholithropine 75UI one vial subcutaneous (SC), daily, for COS, for a total of 11 days, starting on day two of the cycle. We performed the trigger on the 13^th^ day of the cycle, with 0.25mg Alfa coriogonadotropin, one vial in a single dose, SC, 35 hours before follicular puncture. We captured one oocyte in metaphase II (MII), two oocytes in metaphase I (MI), three dysmorphic oocytes and two degenerate oocytes on the 15th day of the cycle. Seminal recovered with 3.0x10^6^sptz/ml, by the Swim-up technique. We performed Intracytoplasmic Sperm Injection (ICSI) on the single MII oocyte. After 18 hours, there were two pronuclei and two polar corpuscles, characterizing fertilization, and after 48 hours, the formation of an embryo of five cells, with 15% fragmentation and cellular asymmetry. We froze the embryo (figure 1) on the 2^nd^ day of development, using the Cryotech method (Gandhi *et al*., 2017).

**Figure 2:**
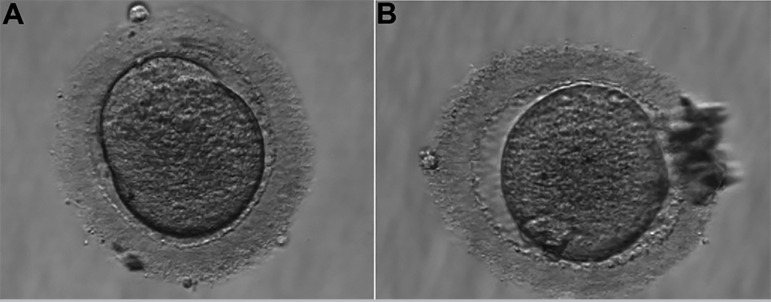
**A.** Oocyte in metaphase I. **B.** Oocyte in MI after *in vitro* maturation, in metaphase II (2^nd^ IVF cycle).

The second IVF cycle occurred in February 2018. The number of antral follicles on the 2^nd^ day of the cycle were 09. Alfa pholithropine/Lutropin Alfa (150UI 75UI) two vials a day, SC, were used for COS, for a total of 10 days. We used cetrorelix acetate 0.25mg, SC, per day, to block the LH peak, started when at least one follicle was greater than or equal to 14mm, for a total of five days of medication. We performed the trigger on the 12th day of the cycle with 0.25mg Alfa coriogonadotropin 01 vial and Triptorelin Acetate 0.1mg/ml, 02 vials, single dose SC, 35 hours before follicular puncture. We captured three MI oocytes (figures 2.a, 3, and 4.a), showing changes in shape, marked cytoplasmic granulation and thick pellucid zone (> 15 microns), on the 14th day of the cycle. After *in vitro* maturation, two oocytes proceeded to MII (figures 2.b and 4.b) and we performed ICSI. Evaluation after 18 hours showed one degenerated and one unfertilized oocyte.

**Figure 3:**
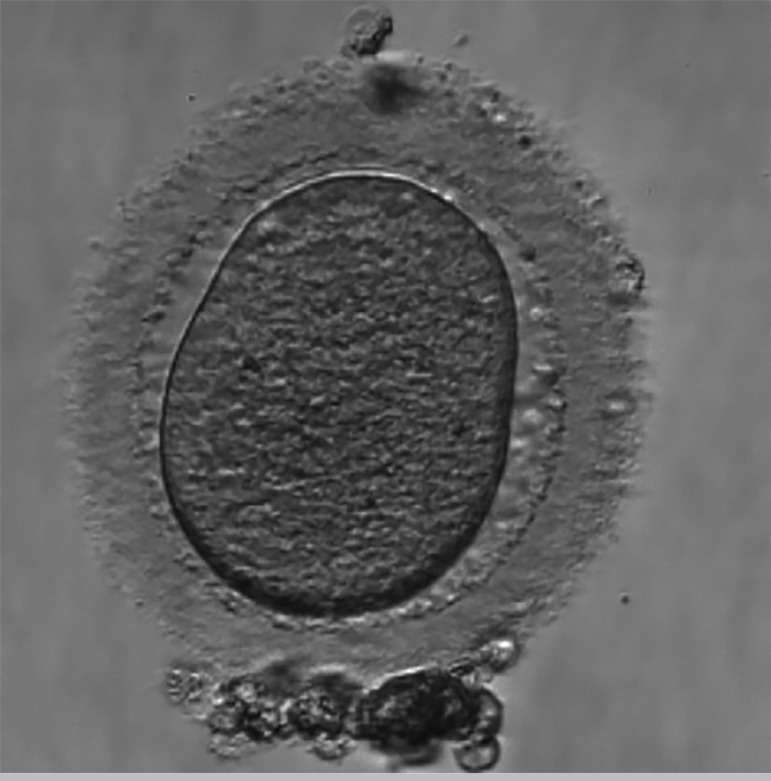
Oocyte in metaphase I that did not develop after *in vitro* maturation (2^nd^ IVF cycle).

**Figure 4:**
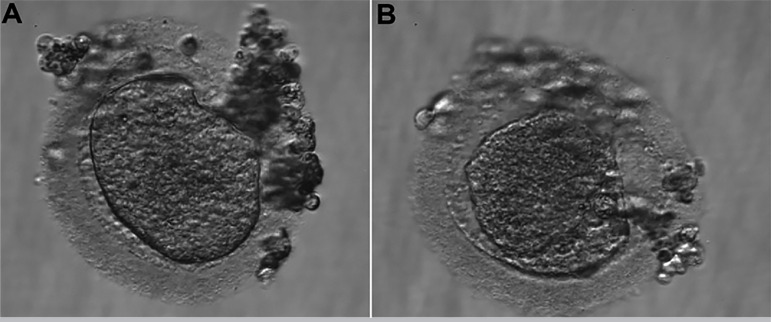
**A.** Oocyte in metaphase I. **B.** Oocyte in MI after *in vitro* maturation, in metaphase II (2^nd^ IVF cycle).

**Figure 5:**
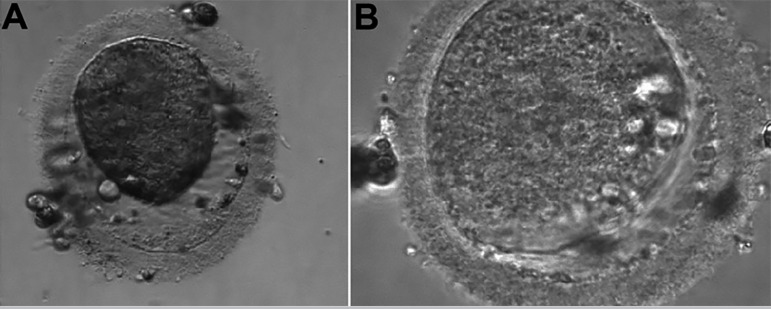
**A.** Oocyte in metaphase II. **B.** Oocyte MII fertilized, with 04 pronuclei and 04 polar body (3^rd^ IVF cycle).

**Figure 6:**
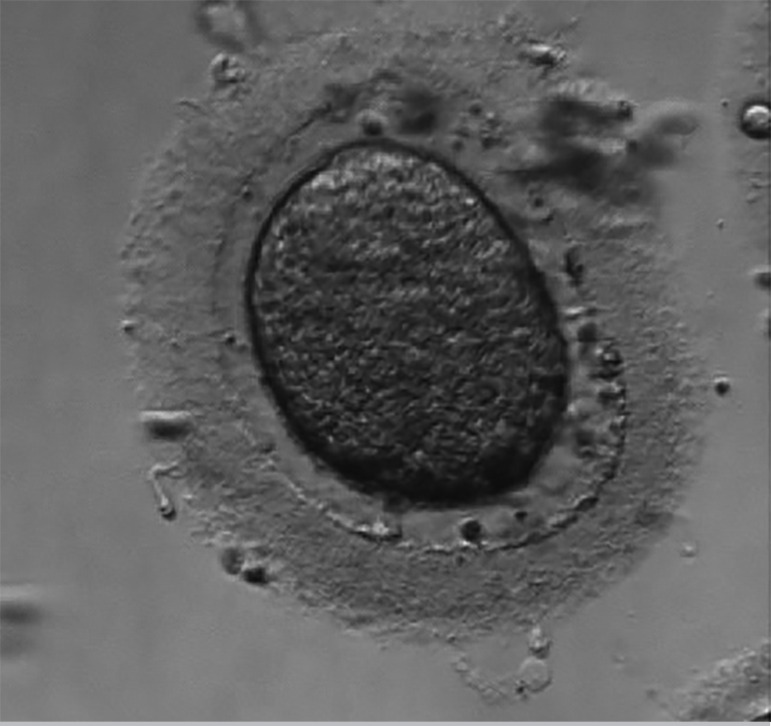
Oocyte 2 in metaphase I, that didn’t develop after *in vitro* maturation (3^rd^ IVF cycle).

**Figure 7:**
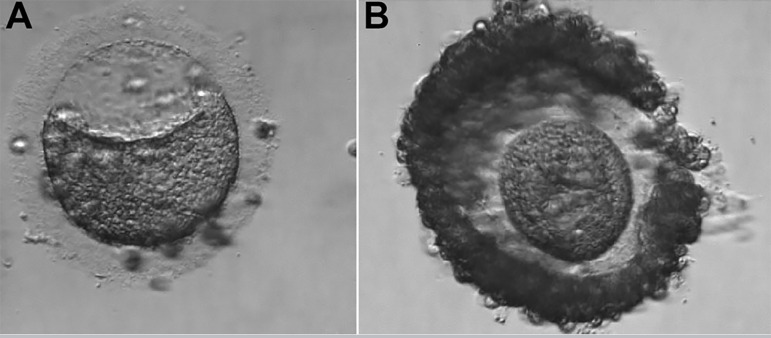
**A.** Dysmorphic oocyte. **B.** Dysmorphic oocyte (3^rd^ IVF cycle).

The third IVF cycle occurred in June 2018. There were 18 antral follicles on the 2^nd^ day of the cycle. For COS, we used Letrozole 5mg daily, orally, from the 2^nd^ to the 6^th^ day of the cycle, and Betafolitropine 300UI a day, SC, associated with Menotropin 75UI a day, SC, from the 2^nd^ day of the cycle, for a total of thirteen days. We used Cetrorelix Acetate 0.25mg, SC, a day to block the LH peak, from the 6th day of the cycle, for a total of 08 days. We performed the trigger on the 15^th^ day of the cycle, with Alfa coriogonadotropin 0.25mg, 01 vial, and Triptorelin Acetate 0.1mg/ml, 02 vials, single dose, SC, 35 hours before the follicular puncture. We captured 4 oocytes on the 17^th^ day of the cycle. One oocyte was in MII (figure 5.a), with reduced cytoplasmic volume, increased cytoplasmic granulation, thick pellucid zone and debris inside, one oocyte was in MI (figure 6), with cytoplasmic granulation and altered shape, and there were two oocytes of unclassifiable maturity (figures 7.a and 7.b), with severe reduction in cytoplasmic volume. We recovered semen with 5.0 x 106 sptz/ml by the Swim-up technique. ICSI was performed on the MII oocyte (figure 5.b), resulting in abnormal fertilization, with the presence of four seedlings after 18 hours of culture media.

In July of 2018, we made a pituitary block due to her adenomyosis, using Leuprolide Acetate 7.5mg, IM, in a single dose, three months before endometrial preparation for embryo transfer of the only one frozen embryo, occurred in January 2018. Endometrial preparation was performed in October 2018, with estradiol valerate 6mg daily, for nineteen days. Micronized progesterone, 600mg a day was started 3 days before the scheduled embryo transfer. Unfortunately, the vitrified embryo did not survive, after thawed by the Cryotech method (Gandhi *et al*., 2017).

**Figure 8:**
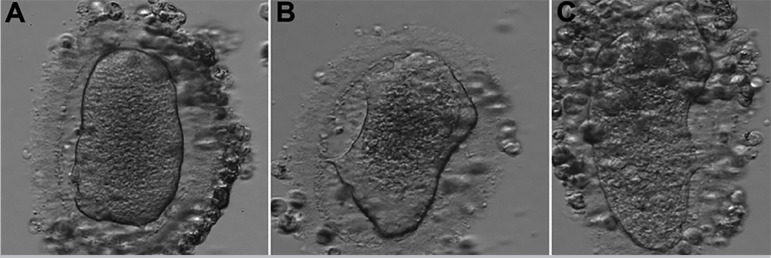
**A.** Oocyte in metaphase I. **B.** Oocyte in prophase I (4^th^ IVF cycle). **C.** Dysmorphic oocyte (4^th^ IVF cycle).

The fourth IVF cycle occurred in July 2019. There were 12 antral follicles on the 2^nd^ day of the cycle. For COS, we used Letrozole, 5mg daily, orally, from the 2nd to the 6th day of the cycle, and Menotropin 150UI daily, SC, from the 4^th^ day of the cycle, for a total of 10 days. We used ganirelix acetate 0.25 mg, daily, SC, to block the LH peak, from the 6th day of the cycle, for a total of four days. We performed the trigger on the 12^th^ day of the cycle, with Human Chorionic Gonadotropin (HCG) 10,000UI, SC, in a single dose, 35 hours before follicular puncture. We captured three oocytes on the 14^th^ day of the cycle. Of them, one oocyte was in MI (figure 8.a), one was in prophase I (PI) (figure 8.b), and one was dysmorphic, with degenerative characteristics (figure 8.c). All of them presented similar morphological characteristics to the oocytes previously produced. The MI oocyte did not progress to MII during the *in vitro* maturation, and the cycle was interrupted due to the absence of viable oocytes. The couple continued to follow up for the egg donation process in 2020.

## LITERATURE REVIEW

### OOCYTE QUALITY

Good oocyte quality includes ideal morphological characteristics, but also its ability to develop, be mature, be fertilized by a sperm and generate a competent embryo that results in a baby alive at home. However, even today, the latter are almost exclusively analyzed from an embryo point of view (Figueira *et al*., 2015; 2010; Setti *et al*., 2011).

Several disorders have the ability to modify the environment around the egg, which may cause changes in its morphology and competence. The follicular fluid produced by the granulosa cells during the development of the secondary follicle, is in close contact with the cumulus-oocyte complex, and both exchange information for the proper development and maturation of the oocyte. Composed by hormonal steroids, metabolites, polysaccharides, proteins, reactive oxygen specimens (ROS), and antioxidants, changes to the follicular fluid components seem to influence oocyte quality, embryo development and pregnancy rates (Da Broi *et al*., 2018). Changes to the substrates of the culture medium, during *in vitro* maturation, have a similar effect on the oocyte and embryo (Dunning *et al*., 2014).

An imbalance in cellular metabolism can result in oxidative stress, which induces apoptosis on oocytes, both *in vitro* and in vivo, and worsens reproductive outcomes in several mammals, including humans. Oxidative stress also triggers a reduction in free androgen production, which favors the apoptosis of granulosa cells and a reduction in estradiol levels, interfering in the communication between granulosa cells and oocytes. As a result, there is a reduction in the supply of nutrients and subtracts that enable the egg maturation process (Prasad *et al*., 2016; Da Broi & Navarro 2016; Meldrum *et al*., 2016). Mitochondria are considered primarily responsible for the development of oocyte competence. Any factor that alters mitochondrial replication or its function can influence oocyte quality. It is believed that the transition from oocyte development control to zygote depends, predominantly, on the components present in the primordial oocytes' cytoplasm or they were acquired during follicular growth. This includes messenger RNA (mRNA), proteins and mitochondria (Meldrum *et al*., 2016). Fresh or mature thawed human oocytes, which have a significant reduction in the number of cytoplasmic mitochondria, develop a defect in the egg´s fertilization by the sperm (Roth, 2018). Studies have shown that stressors, such as maternal nutrition, maternal age, food toxins, inflammatory diseases and heat stress, affect oocyte quality (Roth, 2018).

The woman’s age is a determining factor in oocyte quality. As age increases, there is a reduction in the amount of adrenal androgenic hormones, with a reduction in free circulating testosterone, which, in turn, affects the levels of androgens in the follicular fluid, favoring the reduction of granulosa cells by the apoptosis´ process (Meldrum *et al*., 2016). As a result, there is less production of steroids and glycoproteins, greater exposure to reactive oxygen specimens (ROS), genomic instability of mitochondrial DNA, with rearrangements and deletion of mitochondrial DNA (Keefe *et al.*, 2015; Vollenhoven & Hunt, 2018). The delay in resuming meiosis in elderly women results in increased segregation errors and, consequently, aneuploidies (Keefe *et al*., 2015; Vollenhoven & Hunt, 2018). The consumption of alcohol and cigarettes provides an overproduction of ROS, causing oxidative stress, and negatively affecting fertility (Vollenhoven & Hunt, 2018; Prasad *et al*., 2016; Meldrum *et al*., 2016). Stress, regardless of the triggering factor, is known to alter cortisol levels, with a reduction in estradiol biosynthesis by follicular cells, resulting in a lower number and worse quality of mature oocytes (Prasad *et al*., 2016).

There is enough data in the literature to support the harmful effect of obesity on oocyte quality (Broughton & Moley, 2017; Supramaniam *et al*., 2018). Obese women undergoing ART have an altered intrafollicular environment, with high levels of insulin, triglycerides and inflammatory markers, such as C-reactive protein and lactate (Broughton & Moley, 2017). Fat poisoning causes an increase in ROS and triggers mitochondrial and smooth endothelial reticulum changes, leading to apoptosis (Broughton & Moley 2017; Dunning *et al*., 2014). In these women, there is an increase in leptin, which has a pro-inflammatory action in the follicular fluid, interfering with the steroidogenesis’ process in the granulosa cells (Broughton & Moley, 2017; Snider & Wood, 2019). A study involving patients with Polycystic Ovary Syndrome (PCOS) also shows impairment of oocyte competence, with low pregnancy rates, although in the studies there is an association of PCOS with obesity and metabolic disorders (Broughton and Moley 2017). PCOS exacerbates ovarian inflammation and obesity-dependent oxidative stress (Broughton & Moley 2017; Snider & Wood, 2019).

In endometriosis, heterotopic endometrial cell implants cause an inflammatory reaction, increasing the amount of ROS, with changes in the peritoneal fluid, which compromise folliculogenesis causing oocyte oxidative damage (Da Broi & Navarro, 2016). In the *in vitro* fertilization (IVF) cycles, during a COS, there is oocyte recruitment, which naturally would not advance to a metaphase II. As a result, compromised-quality oocytes become more frequent, interfering with the fertilization and embryo development rates (Rienzi *et al*. 2011; Alpha Scientists in Reproductive Medicine & ESHRE Special Interest Group of Embryology, 2011). However, establishing a direct relationship between COS and oocyte quality is difficult, since the most commonly used parameter to analyze this outcome is the pregnancy rate, which is influenced by several other factors (Bosch *et al*., 2016). Although ovarian stimulation alters the functionality of follicular cells and several studies report a deleterious, dose-dependent, effect of gonadotropins on egg and embryo quality, these evidences are not supported when comparing natural cycles with IVF cycles (Bosch *et al*., 2016).

Many studies have shown the relationship of genetic polymorphism, in FSH (FSHR) and LH (LHR) receptors, estrogen receptors (ER) and anti-Mullerian hormone receptors (AMHR), and the response to exogenous gonadotropins used during COS. However, most of these studies analyze the response to gonadotropins based on the number of oocytes collected, number of mature oocytes and blastocysts formed, but not on the morphological and functional aspects of the egg (Čuš *et al*., 2019; Alviggi *et al*., 2018).

## OOCYTE MORPHOLOGY

An excellent oocyte morphology has a spherical structure, surrounded by a uniform pellucid zone, with a homogeneous and translucent cytoplasm, free of inclusions, and with an appropriately sized polar corpuscle (Alpha Scientists in Reproductive Medicine & ESHRE Special Interest Group of Embryology, 2011). However, for this to happen the oocyte must mature, which can be understood as a sequence of events, that begins with follicular development, steroidogenesis and changes to the intra-follicular environment, followed by cytoplasmic development, with mRNA transcription and protein formation, ending with nuclear maturation (Sanchez *et al*. 2017; Setti *et al*., 2011; Rienzi *et al*., 2011). To form a haploid oocyte, two-cell division must occur, meiosis I and meiosis II (Touati & Wassmann, 2016; Keefe *et al*., 2015; Figueira *et al*., 2015). The onset of meiosis I in women occurs in the fetal period, with some oocytes remaining in this stage for long periods, in the germinal vesicle´s form. This seems to contribute to the occurrence of aneuploidies. The oocytes remain at the beginning of meiosis I, asleep, until the puberty phase, when only a few will pass to the meiosis II stage (Touati & Wassmann, 2016). Meiosis I ends with the LH peak, in the middle of the menstrual cycle, and the separation of homologous chromosomes. The extrusion of the 1^st^ polar corpuscle during metaphase II (MII) of meiosis II is the marker of oocyte maturity (Figueira *et al*., 2010). In meiosis II, the sister chromatids are separated, generating haploid oocytes (Keefe *et al*., 2015; Touati & Wassmann, 2016). After oocyte fertilization, the 2^nd^ polar body is extruded (Vollenhoven & Hunt, 2018; Touati & Wassmann, 2016). The cytoplasmic and nuclear maturation are not the same process, and they are not necessarily synchronous with each other (Alpha Scientists in Reproductive Medicine & ESHRE Special Interest Group of Embryology, 2011).

Van Blerkom and Henry initially described oocyte dysmorphism in 1992. At this time, dysmorphic oocytes did not fertilize by the conventional IVF technique, and nowadays, with the ICSI technique, they could, with apparently normal development in the early stages of the embryo formation. However, the high rates of embryo loss up to the blastocyst stage reinforces that defects inherent in the oocyte can compromise the subsequent embryo development (Alpha Scientists in Reproductive Medicine & ESHRE Special Interest Group of Embryology, 2011; Figueira *et al*., 2010).

In order to improve the reproducibility data of oocyte and embryo analysis, the Istanbul Consensus divided oocyte anomalies into intra and extra cytoplasmic (Alpha Scientists in Reproductive Medicine & ESHRE Special Interest Group of Embryology, 2011). The oocyte score proposed is based on the analysis of these characteristics. From intracytoplasmic aspects, we evaluate the texture of the cytoplasm, which should be homogeneous and without granulations; the presence of organelle clusters; and the presence of vacuoles. Organelle clusters reduce implantation rates in these oocytes (Alpha Scientists in Reproductive Medicine & ESHRE Special Interest Group of Embryology, 2011). The smooth endothelial reticulum (SER) cluster is relatively rare in oocytes recruited by different types of COS, and its importance lies on the fact that it can cause dysmorphic phenotypes, due to imprinting disorders in newborns, such as Beckwith-Wiedemann syndrome. This change is also associated with early pregnancy loss. The presence of cytoplasmic vacuoles larger than 14 µm in diameter is related to fertilization failures and reduction in blastocyst rates. From the extra cytoplasmic aspects, the cumulus-oocyte complex is evaluated in a binary relationship with I in the presence of expanded cumulus and corona radiata present; the pellucid zone as to its color and thickness; and the perivitelline space, if it is extremely wide. The presence of inclusions in the perivitelline space is considered an abnormality that should be noted, but its importance in the reproductive outcome is still unclear. The presence of the 1^st^ polar corpuscle is also evaluated, and when it has an abnormally enlarged size, this oocyte should not be fertilized due to the high risk of aneuploidy (Alpha Scientists in Reproductive Medicine & ESHRE Special Interest Group of Embryology, 2011). In general, the size and number of oocyte anomalies is directly related to pre-implantation developmental failures (Alpha Scientists in Reproductive Medicine & ESHRE Special Interest Group of Embryology, 2011).

Still on oocyte morphology, a meta-analysis carried out in 2011 (Setti *et al*., 2011) corroborates the recommendations of the Istanbul Consensus, by reporting that the presence of an increased polar corpuscle, an increased perivitelline space, the presence of retractable bodies or cytoplasmic vacuoles, reduce the rate of fertilization. A systematic review published in the same year (Rienzi *et al*., 2011), on different morphological parameters used in oocyte analysis, identified 50 studies, of which 33 of them showed a significant correlation between oocyte morphology and ART outcomes. Although the conclusion of the systematic review does not identify a parameter with a strong predictive value for reproductive outcomes, some findings need to be mentioned. There was a relationship between a very dense radiating corona and a reduction in oocyte maturity, the presence of central granulations and negative effect on the fertilization rate and blastocysts formation. A thicker pellucid zone and better embryo quality and a higher bio refringence of the pellucid zone with improvements in implantation and clinical pregnancy rates. A low bio refringence of the pellucid zone and increased pregnancy loss were also related (Rienzi *et al.,* 2011). Giant oocytes seem related to chromosomal polysomes (Setti *et al*., 2011; Alpha Scientists in Reproductive Medicine & ESHRE Special Interest Group of Embryology, 2011; Rienzi *et al*., 2011). Cytoplasmic vacuoles worsen the frozen oocytes’ survival and the development of competent embryos after fertilization, and it is, therefore, associated with lower rates of clinical pregnancy and higher rates of chemical pregnancy. Cytoplasm viscosity and cell membrane resistance have a deleterious effect on fertilization, embryo quality, blastocyst and fertilization rates, respectively (Rienzi *et al*., 2011).

Oxidative stress, caused by systemic disorders, is responsible for stopping the cell cycle and stimulating apoptosis in mature and immature oocytes; however, immature oocytes seem to be more susceptible to morphological changes such as shrinkage, membrane defects, cytoplasmic granulations and degeneration (Prasad *et al*., 2016). The excess of fatty acids in the follicular fluid of obese women causes morphological changes in the cumulus-oocyte complex (Broughton & Moley; 2017). Immature oocytes derived from women with endometriosis showed a reduction in the cortical cell layer, and a more rigid pellucid zone, making the hatching and embryo implantation a difficult process (Goud *et al*., 2014). Two other studies showed that changes to oocyte staining, granular or dark cytoplasm, defective mitochondria, extrusion or incomplete division of the 1^st^ polar corpuscle, loss of the cortical layer, and break of the cell spindle were more frequent in patients with endometriosis (Kasapoglu *et al*., 2018; Ceviren *et al*., 2014). A study carried out on MII oocytes, involving patients with mild to moderate endometriosis, reported the presence of chromatin decentralization and large nucleoli, a lower number of mitochondria, and mitochondrial abnormalities (Xu *et al*., 2015).

The meiotic spindle is responsible for the correct chromosomal reduction and a haploid oocyte formation. Its analysis can be performed through a polarized light microscope, and it appears to predict outcomes of embryo formation, rather than implantation rate and clinical pregnancy (Keefe *et al*., 2015). Advanced woman’s age causes changes to the formation of the meiotic spindle and chromosomal alignment, which results in higher rates of aneuploidy (Vollenhoven & Hunt, 2018). In women over 40 years of age, 80% of oocytes exhibit changes in the meiotic spindle and/or chromosomal alignment (Keefe *et al*., 2015). The shortening of telomeres and instability in their cohesion can cause rupture of the chiasma and meiotic spindle, and increase the ROS concentration (Keefe *et al*., 2015; Vollenhoven & Hunt, 2018). A study on oocytes from women with morbid obesity, who were not fertilized in ART, showed meiotic spindle disorders with chromosomal misalignment, similar to those described in rats (Broughton & Moley, 2017).

## FINAL CONSIDERATIONS

Given that many lifestyle conditions are responsible for changes to oocyte quality and morphology, some habit changes should be encouraged. Suspension of alcohol consumption during treatment is beneficial in reproductive outcomes (Prasad *et al*., 2016; Meldrum *et al*., 2016). Smoking cessation for a period of 3 to 6 months seems to be beneficial (Meldrum *et al*., 2016). Improving diet, choosing foods rich in antioxidants such as pomegranate, chocolate, licorice, cumin, ginger, oregano, fruits, vegetables and fish can all help reduce free radicals. The use of antioxidant supplements such as vitamin C, vitamin E, Zinc, Selenium, Omega 3 also seems to help balance the oxidative process (Prasad *et al*., 2016; Meldrum *et al*., 2016). All obese patients, but especially those with a BMI greater than or equal to 35 kg/m^2^, should be encouraged to lose weight before starting IVF cycles, especially those under 38. Exercise is important in the slimming process (Meldrum *et al*., 2016).

Research of an ideal predictor of oocyte quality are ongoing. Preimplantation genetic analysis of the polar corpuscle has been studied by some authors, using the array comparative genomic hybridization (aCGH) methodology, and has shown to be predictive of embryo aneuploidy; however, some oocytes with aneuploid polar corpuscle have generated euploid embryos. This possible mechanism of oocyte self-correction is unclear, but it reduces the specificity of pre-implantation analysis of the polar corpuscle of the oocyte (Keefe *et al*., 2015). The telomere’s analysis of the polar corpuscle seems to be a promising marker of oocyte quality, insofar as it seems to have a more reliable relationship with embryo aneuploidy (Keefe *et al*., 2015). The use of time-lapse technology, which is more and more frequent, can bring accurate information about the predictive value of oocyte morphology to reproductive outcome in the future (Rienzi *et al*., 2011).

Given the above, the morphological aspect of the oocyte is multifactorial, making it difficult to find a unique response to poor oocyte quality.

## CONCLUSION

The case report above shows a patient who, despite being 36 years old, having a good ovarian reserve identified by the AFC present at the beginning of each stimulated cycle, showed, in all of her oocytes captured, some degree of dysmorphism, often with more than one abnormality. Regardless of the protocol, dose of gonadotropin, type of gonadotropin used, and stimulation days, there was no change to oocyte pattern, reinforcing the relationship between oocyte morphology and embryo outcome. Although the exact mechanism concerning the relationship between oocyte morphology and reproductive outcomes remains under study, the deepening of this knowledge can assist physicians in decision-making, in cases of poor response to ART, since it has a low cost and easy access in human reproduction laboratories. Future studies with adequate methodology are important for the real assessment between oocyte morphology, embryonic development, dose and medication formulation and live birth rate.
